# Pharmacokinetic Comparison of Epinastine Using Developed Human Plasma Assays

**DOI:** 10.3390/molecules25010209

**Published:** 2020-01-03

**Authors:** Seung-Hyun Jeong, Ji-Hun Jang, Hea-Young Cho, Yong-Bok Lee

**Affiliations:** 1 College of Pharmacy, Chonnam National University, 77 Yongbong-ro, Buk-Gu, Gwangju 61186, Korea; rhdqn95@naver.com (S.-H.J.); jangji0121@naver.com (J.-H.J.); 2 College of Pharmacy, CHA University, 335 Pangyo-ro, Bundang-gu, Seongnam-si, Gyeonggi-Do 13488, Korea; hycho@cha.ac.kr

**Keywords:** epinastine, pharmacokinetics, HPLC-UV, UPLC-MS/MS, comparison

## Abstract

The purpose of the study was to develop two new methods, HPLC-UV and UPLC-MS/MS, for quantifying epinastine in human plasma and to compare pharmacokinetic (PK) parameters obtained using them. Even in the same sample, there may be a difference in the quantitative value of drug depending on the assay, so that minor changes in PK parameter values may affect drug dose and usage settings. Therefore, selection and establishment of analytical methods are very important in PK studies of drugs, and a comparison of PK parameters according to analytical methods will be vital. For this study of PK parameter change, we newly developed two methods, HPLC-UV and UPLC-MS/MS, which are most commonly used to quantify epinastine concentrations in human plasma. All developed methods satisfied the international guidelines and criteria for successful application to PK study of 20 mg epinastine hydrochloride tablets after oral administration to twenty-six humans. A comparison of these two methods for in vivo analysis of epinastine was performed for the first time. This comparison study confirmed that different dose and usage settings might be possible based on PK parameters calculated using other analyses. Such changes in calculated PK parameters according to analytical methods would be crucial in the clinic.

## 1. Introduction

Epinastine is a histamine H_1_ receptor antagonist with high receptor selectivity. Epinastine cannot cross the blood-brain barrier in the body and may be classified as a second-generation antihistamine [1]. Based on its polarity and cationic charge at physiological pH, it cannot easily diffuse into the central nervous system (CNS) [2]. Thus, these physicochemical properties distinguish epinastine as a non-sedative antihistamine, and unlike other first-generation antihistamines acting on the CNS, it does not cause side effects, such as drowsiness or sedation [3]. Epinastine has multiple effects that inhibit the allergic response in three ways: (1) stabilizes mast cells by preventing mast cell degranulation to control the allergic response, (2) prevents histamine binding to both the H_1_- and H_2_-receptors to stop itching and provide lasting protection, and (3) prevents the release of pro-inflammatory chemical mediators from the blood vessel to halt progression of the allergic response [4]. In addition, Oshima et al. [5] reported that epinastine suppresses the immune responses of Th-2 cell through functional modulation of dendritic cells, which play an essential role in the development of allergic immune responses, and thus has the results in favorable modification of the clinical status of allergic diseases. Epinastine has been used in the form of eye drops primarily in association with allergic conjunctivitis. In this regard, various safety information has been established through previous preclinical and clinical trials. Brar et al. [6] reported that no significant ocular and systemic toxicity was observed in white rabbits and cynomolgus monkeys treated with 0.05–0.5% epinastine hydrochloride ophthalmic solution for 6 months. Yu et al. [7] reported that epinastine rapidly reached high levels on the ocular surface without unwanted systemic side effects when 0.05% epinastine hydrochloride ophthalmic solution was administered to allergic conjunctivitis patients for 7 days. In addition to its use as eye drops, during the last 10−15 years, tablets of epinastine hydrochloride have been used for clinical application in asthma and urticaria, and patients are prescribed at a dose of 10−20 mg once daily. In vivo metabolism of epinastine occurs mainly in the liver, but the degree of metabolism is reported to be very low. That is, most of the epinastine administered into the body is excreted in the unchanged form [8].

Analytical and pharmacokinetic (PK) reports of epinastine have been performed in the past. However, the reported methods had limitations and needed improvement. Most of all, there have been no reports on how PK characteristics can differ by analytical methods, and which PK parameters are significantly affected. Even in the same sample, there may be differences in the quantitative values depending on the analytical method, which may cause differences in the PK parameter values. Drug dose and usage settings based on the individual parameters for which the difference occurred may produce different results in the clinic. Therefore, in this study, we tried to identify the changes in PK parameters of the drug according to the analytical methods. For this study, epinastine hydrochloride tablets of 20 mg were orally administered to humans, and the subsequently collected plasma samples were analyzed by two newly developed assays (HPLC-UV and UPLC-MS/MS), which are most commonly used for quantification in biological samples. Our study also focused on comparing the performances of newly developed UPLC-MS/MS and HPLC-UV methods for the determination of epinastine in human plasma. All methods were fully validated according to international bioanalytical method guidelines and represent a useful tool for the characterization of PKs of 20 mg epinastine hydrochloride tablets in Korean subjects.

## 2. Results

### 2.1. Method Development

#### 2.1.1. UPLC-MS/MS Method

**Figure 1:**
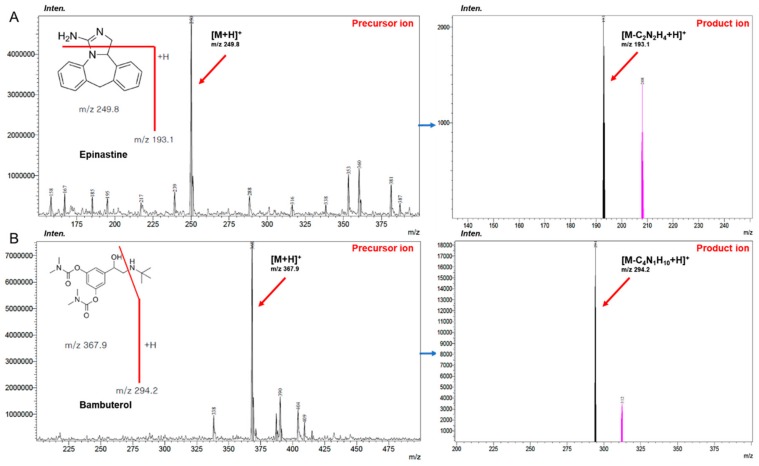
Positive product ion mass spectra in UPLC-MS/MS quantification; (**A**) epinastine; (**B**) bambuterol (IS). IS: internal standard.

In this study, an improved accurate and sensitive UPLC–MS/MS method was newly developed for determining epinastine in human plasma. The product ion mass spectra of epinastine were obtained by scanning the individual standard solution into the mass spectrometer. Epinastine generated protonated molecular ion as [M+H]^+^ in positive-ion mode. Internal standard (IS) was also performed in scan mode similarly and generated a protonated molecule ion of [M+H]^+^, such as epinastine. The most abundant fragment ion for multiple reaction monitoring (MRM) was *m*/*z* 249.8 → 193.1 for epinastine and *m*/*z* 367.9 → 294.2 for the IS, respectively. The mass spectra results of epinastine were the same as those of Do et al. [9] and Bae et al. [10]. Figure 1 presents the relevant mass spectra. Other ionization parameters that optimized the quantification of epinastine were desolvation temperature of 250 °C, collision energy of −36 eV, nebulizing nitrogen gas flow of 3 L/min, and drying nitrogen gas flow of 15 L/min.

**Figure 2:**
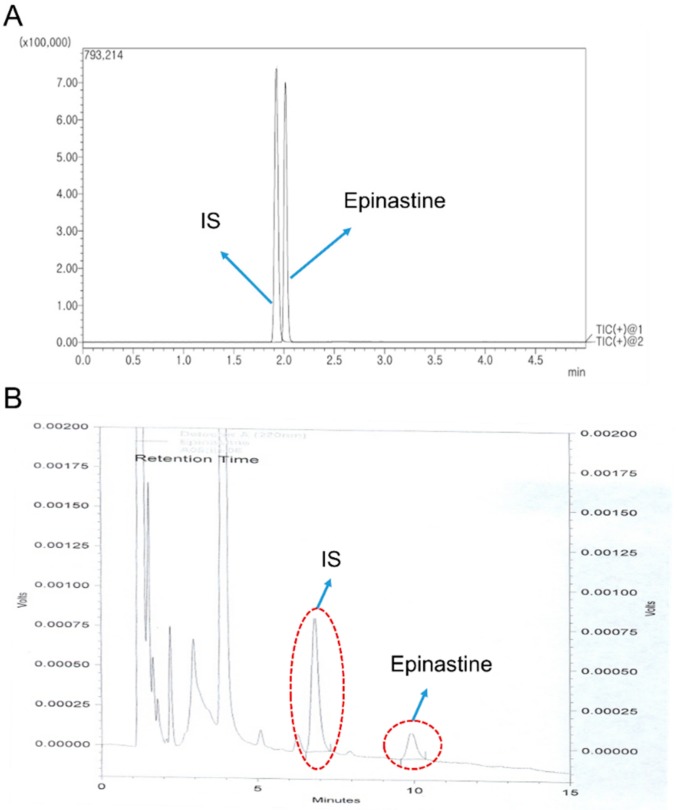
Representative chromatograms of epinastine and IS in MRM (multiple reaction monitoring) positive mode of UPLC-MS/MS (**A**) and HPLC-UV (**B**) method in human plasma.

A mobile phase with various organic solvents (including precipitation and extraction solvents), pH, and different columns was tested to yield high resolution and adequate sensitivity. The Acquity UPLC^®^ BEH C_18_, HALO-C_18_, and Phenomenex KINETEX core-shell C_18_ column using acetonitrile (ACN) and 0.1% (*v*/*v*) aqueous formic acid containing 1% (*v*/*v*) of 5 mM ammonium formate (pH 3.0) buffer by gradient elution at a flow rate of 0.3 mL/min were tested to obtain an optimum chromatogram. Although the three columns mentioned above have the same chemical structure (octadecyl, C_18_) as the stationary phase, the charging technique differs slightly from each manufacturer and also varies in particle size. Finally, the Phenomenex KINETEX core-shell C_18_ column (50 × 2.1 mm, 1.7 μm particle size) was selected because it showed relatively good symmetric peak shapes, selectivity, and sensitivity for epinastine quantification. We used a mobile phase with ACN and water containing 0.05% (*v*/*v*) trifluoracetic acid [11] or 0.3% (*v*/*v*) triethylamine [12] with Phenomenex KINETEX core-shell C_18_ column at a flow rate of 0.3 mL/min by gradient elution for epinastine quantification. However, these results were unsatisfactory for chromatograms of epinastine due to low sensitivity and incomplete peak symmetry. To overcome these problems, we additionally tested with 1% (*v*/*v*) of 5 mM ammonium formate (pH 3.0) buffers of varying pH, which included 0.1% acetic acid in water (final pH 2.9), 0.01% formic acid in water (final pH 2.7), and 0.05% formic acid in water (final pH 2.5), as well as 0.1% formic acid in water (final pH 2.3), which displayed the best resolution and highest intensity. In addition, ACN as mobile phase B showed a better resolution and a higher sensitive response than the mobile phase containing methanol reported in other studies [12]. As a result, water containing 0.1% formic acid containing 1% (*v*/*v*) of 5 mM ammonium formate (pH 3.0) buffer (mobile phase A, pH 2.3) and ACN (mobile phase B) were selected as mobile phases using gradient elution program. The gradient elution was satisfactory regarding the retention time, peak shape, and interference peaks separating epinastine. In addition, 1% (*v*/*v*) of 5 mM ammonium formate buffer (pH 3.0) maintained the retention time of epinastine constant. Figure 2A presents a representative MRM chromatogram with moderate retention times of 2.07 min for epinastine and 1.99 min for the IS.

Protein precipitation (PP) and liquid-liquid extraction (LLE) methods for sample extraction were tested. For the LLE method, di-ethyl ether, ethyl acetate, methylene chloride, and methyl tert-butyl ether (MTBE) were used for extraction from the sample. For epinastine, ethyl acetate showed much better extraction efficiency than methylene chloride, di-ethyl ether, and MTBE. Furthermore, the PP method using ACN and methanol was also tested. Methanol showed a lower noise and higher sensitive response than ACN and was finally selected as precipitation solvent for the PP method. Therefore, the PP method with methanol and the LLE method with ethyl acetate were optimized for the determination of epinastine in human plasma samples. In addition, the supernatant after extraction was evaporated to dryness under a gentle nitrogen stream at 35 °C to improve the sensitivity of epinastine in this UPLC-MS/MS method. This newly developed UPLC-MS/MS method provided higher sensitivity compared with the previous study in which the lower limit of quantitation (LLOQ) value was 1 ng/mL [10], with an LLOQ of 0.02 ng/mL.

#### 2.1.2. HPLC-UV Method

A reversed-phase Nova-Pak C_18_ column (150 × 3.9 mm, 4 μm particle size) was used in the HPLC-UV method. The peak symmetry, selectivity, and shape of epinastine in the mobile phase, consisting of 20 mM phosphate (pH 5.6) buffer and methanol mixed with a small amount of ACN (64/30/6, *v*/*v*/*v*) at a flow rate of 0.8 mL/min, were observed. Figure 2B presents representative HPLC chromatograms with moderate retention times of 6.82 min for the IS and 9.91 min for epinastine. The HPLC-UV method was conducted using the isocratic elution, and the total run time was 15 min per sample. Bambuterol was selected as the IS based on peak shape and retention time in the selected column and extraction efficiency in human plasma. Only the LLE method with dichloromethane was optimized for quantification of epinastine in the sample preparation process, based on a reference related to epinastine analysis [13]. In this process, epinastine was a weakly basic substance and existed as cation at the biological pH. Thus, the plasma sample was treated with 500 μL of 0.1 M sodium carbonate to increase the extraction efficiency to the organic layer. The back-extraction process of epinastine into the aqueous layer was carried out using a 200 μL of 25 mM sulfuric acid solution. The HPLC-UV method used centrifuged upper layer directly without concentrating the decompression evaporation method using nitrogen, unlike the UPLC-MS/MS method. As a result, the time and steps required for sample preparation were reduced.

### 2.2. Quantitative Method Validation

#### 2.2.1. Selectivity

**Figure 3:**
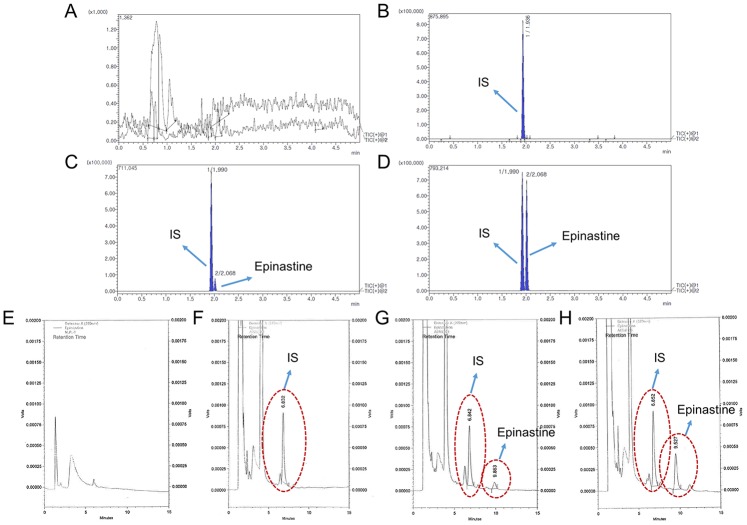
Chromatograms of blank plasma (**A** and **E**), zero plasma containing IS (**B** and **F**), blank plasma containing LLOQ (lower limit of quantitation) of epinastine and IS (**C** and **G**), the plasma sample at 2 h after oral administration of 20 mg epinastine hydrochloride tablet (**D** and **H**); A–D, UPLC-MS/MS; E–H, HPLC-UV.

Selectivity was shown in the response of the blank human plasma (Figure 3A,E), zero plasma containing the IS (Figure 3B,F), blank plasma containing LLOQ epinastine and IS (Figure 3C,G), and the plasma sample at 2 h after oral administration of epinastine hydrochloride tablet (Figure 3D,H). The representative chromatograms are presented in Figure 3, indicating no significant interferences from the endogenous substances around the retention times of analytes in blank plasma in both methods. 

#### 2.2.2. Calibration Curves

Linearity for epinastine in human plasma was excellent over the concentration range of 1–100 ng/mL for HPLC-UV, and 0.02–100 ng/mL for UPLC-MS/MS. All calibration curves fitted well, with the correlation coefficient (r^2^) of 0.99 or more. The linear regression equations of epinastine in human plasma were as follows: *y* = (0.0262 ± 0.0083)*x* + (0.00299 ± 0.00095) for HPLC-UV, and *y* = (0.1478 ± 0.0307)*x* + (0.00263 ± 0.00058) for UPLC-MS/MS with *y* as peak-area ratio of epinastine-to-IS and *x* (ng/mL) as plasma concentration of epinastine. Calibration curves for epinastine in human plasma are shown in Figure S1. The HPLC-UV and UPLC-MS/MS assays yielded LLOQs of 1 ng/mL and 0.02 ng/mL, respectively, suitable for PK study after oral administration of epinastine hydrochloride tablets in humans. The previously conducted HPLC-UV analysis [12] showed that the maximum plasma concentration (C_max_) value of epinastine was not high (approximately mean 10 ng/mL), and was quantified below LLOQ in specific samples that corresponded to the early phase of drug absorption and terminal phase of elimination. Thus, lowering LLOQ was required for identifying a clear PK pattern in the body. The most sensitive LC-MS/MS method reported until now showed an LLOQ of about 1 ng/mL [10]. In comparison, our method had at least 50 times lower LLOQ than the previous method [10].

#### 2.2.3. Accuracy and Precision 

During the validation process of the newly developed method, excellent performance with consistent accuracy and low deviation was observed from four quality control (QC) samples. Table 1 describes the inter- and intra-batch accuracy and precision for epinastine. The intra-batch accuracy ranged from 95.15%−110.23% with a precision (coefficient of variation, CV) of <12.52% in both methods. In addition, the inter-batch accuracy ranged from 93.93–114.47% with a precision (CV) of <8.23% in both analyses. All accuracies were within the range of 93.93% to 114.47%, and CV values for epinastine ranged from 1.32% to 12.52%. As a result, these indicated that both HPLC-UV and UPLC-MS/MS methods were reproducible and accurate for the quantification of epinastine in human plasma.

#### 2.2.4. Matrix Effect and Recovery

Table 2 presents the matrix effect and/or recoveries for epinastine in HPLC-UV and UPLC-MS/MS method. The extraction recoveries of epinastine from human plasma were 91.58–94.36% and 95.93–97.62%. The recoveries of IS were 96.94 ± 4.73% and 99.46 ± 3.54% in HPLC-UV and UPLC-MS/MS analysis, respectively. Significant matrix effects were not seen in the quantification of epinastine (95.28–96.63% with UPLC-MS/MS) or IS (97.35 ± 3.16% with UPLC-MS/MS). The results suggested that the recoveries of the analyte were reproducible, consistent, and precise. The simple LLE and/or PP methods optimized for extracting epinastine from human plasma were successfully applied to the determination of epinastine in human plasma.

#### 2.2.5. Stabilities

In both methods, the stability for epinastine was evaluated using two different levels of QC samples under various conditions. The stabilities were examined as freeze-thaw, short-term, and long-term stability. The results are presented in Table 3. Epinastine was stable in human plasma for 24 h at 25 °C without any significant degradation (range of 97.91–100.35% in both methods). In the long-term stability test for 4 weeks at −80 °C, epinastine was also stable, which guaranteed the quality of quantification after sample collection within 4 weeks (range of 98.42–101.53% in both methods). All analytes were stable after three cycles (range of 96.04–101.62% in both methods) in the freeze-thaw cycle test. Furthermore, epinastine was stable for 24 h at 25 °C ranged from 98.13–101.91% in the HPLC-UV method and stable for 24 h at 15 °C ranged from 99.33–102.09% in UPLC-MS/MS method as post-preparative stability. These ranges were within the limits of the Food and Drug Administration (FDA) guidelines (±15%) in all stability tests. In addition, the stock solutions for IS and epinastine were stable in the storage concentration for 4 weeks at −20 °C. The stability of the epinastine stock solution was 100.14 ± 2.06%, and the stability of the IS stock solution was 98.72 ± 3.38%. All these results suggested the stability of epinastine under various storage conditions.

#### 2.2.6. Carryover

As presented in Figure 3A,E, after injecting the highest concentration (epinastine of 100 ng/mL) sample of the standard curve, no peak of the analytes was shown in the blank plasma sample. It was validated that the carryover had no effect on the analysis.

#### 2.2.7. Incurred Sample Reanalysis

Thirty-two (10% of the total analyzed samples) human plasma samples were examined to evaluate incurred sample reanalysis (ISR). The variability of all samples was within 20% between the value of initial analysis and that of reanalysis in both methods. In addition, twenty-five samples were within 10% in HPLC-UV, and twenty-eight samples were within 10% in UPLC-MS/MS. As a result, the newly developed method showed the reproducibility of the initial analysis results.

### 2.3. Methods Comparison

**Figure 4:**
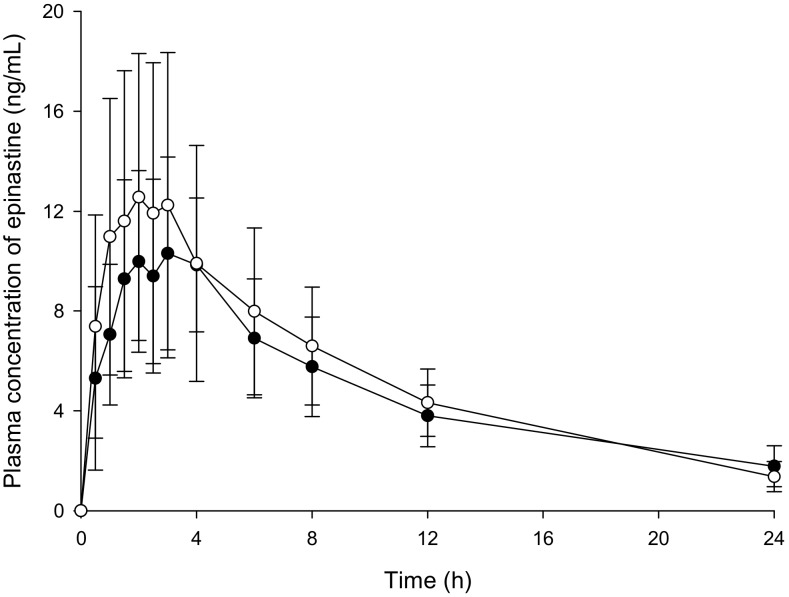
Mean plasma concentration-time profiles of epinastine after oral administration of 20 mg epinastine hydrochloride tablet based on UPLC-MS/MS (-○-) and HPLC-UV (-●-) methods. Vertical bars represent the standard deviation of the mean (n = 26).

Individual HPLC-UV and UPLC-MS/MS methods were developed for the quantification of epinastine in human plasma. Table 4 summarizes the information for both methods. The obtained plasma concentration-time profiles (Figure 4) and calculated PK parameter values (Table 5) for epinastine showed similar findings in HPLC-UV and UPLC-MS/MS methods.

**Figure 5:**
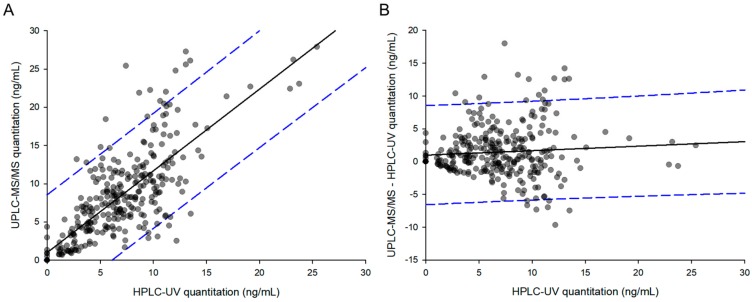
Comparative analysis of samples using HPLC-UV (*x*-axis) and UPLC-MS/MS (*y*-axis) (**A**), and correlation of the method differences between HPLC-UV (*x*-axis) and UPLC-MS/MS – HPLC-UV (*y*-axis) (**B**). The straight line represents the linear regression line, and the dashed line shows a 95% prediction interval line.

Figure 5A depicts a two-dimensional graph of parameters derived from the same human samples. The linear regression line of the graph was as follows: y = 0.99824x + 0.59482. The slope of the regression line was close to one, and the correlation coefficient (r^2^) was 0.84, indicating a strong and reliable correlation. Twenty-two samples (7.05% of total) were measured below LLOQ in HPLC-UV, while they were all quantified in UPLC-MS/MS. These results were plotted on the graph as points on the *y*-axis ranging from 0.07–5, with the *x*-axis being 0. Among the total 312 points, 297 were within the 95% prediction interval (PI), corresponding to a statistical value of 95.19%. Figure 5B shows the difference between the two analytical methods (UPLC-MS/MS-HPLC-UV), depending on the concentrations measured by HPLC-UV. The linear regression line of the graph was as follows: y = 0.0355x + 0.0926. The slope of the regression line was close to 0, indicating that the difference between the two methods was not significant. At concentrations above 15 ng/mL quantified by HPLC-UV, there were no points where the difference between the two methods exceeded the 95% PI. All points outside the 95% PI were observed at less than 15 ng/mL. These results meant that the difference between the two analytical values was greater at lower concentrations than at higher concentrations.

### 2.4. Pharmacokinetic Studies

The newly developed HPLC-UV and UPLC-MS/MS methods were supported in the PK study of epinastine after oral administration of 20 mg epinastine to twenty-six healthy Korean subjects. The concentration-time curves of epinastine in humans using HPLC-UV and UPLC-MS/MS quantification methods are displayed in Figure 4. After oral administration of epinastine hydrochloride tablets, the time to reach C_max_ (T_max_) of epinastine was defined as 2−3 h. Epinastine was rapidly absorbed into the blood and was slowly removed from the blood, which could be detected even 24 h after administration. In addition, the C_max_ was relatively low on the average of 10−20 ng/mL. This might be related to reports that the absolute oral bioavailability of epinastine was as low as about 40%, and that a significant amount of orally administered epinastine was detected in feces rather than urine [1,14]. The estimated PK parameters, including area under the curve (AUC_0–∞_), half-life (t_1/2_), clearance (CL/F), area to final measured concentration (AUC_0–t_), T_max_, C_max_, and volume of distribution (V_d_/F), are presented in Table 5. The t_1/2_ of epinastine reported by Li et al. [15] was 7.79–10.68 h, and the T_max_ was 1.88–2.3 h. The t_1/2_ of epinastine reported by Shi et al. [16] was 8.8–10.4 h, and the T_max_ was 2.0–2.7 h. These were similar to our PK results. The remaining reported PK results were limited for comparison with our results, as they were either in rats or administration of eye drops to the ocular pathway.

### 2.5. Pharmacokinetic Parameters Comparison

The student’s *t*-test was applied to compare the differences between the PK parameters determined by quantification methods of HPLC-UV and UPLC-MS/MS. As a result, the PK parameters calculated by HPLC-UV and UPLC-MS/MS methods were not statistically different (*p* > 0.05). The ratios of AUC_0–∞_ (including AUC_0–t_) and CL/F were close to one between HPLC-UV and UPLC-MS/MS method, whereas the ratios of t_1/2_ and V_d_/F varied depending on the method probably due to the low concentration of the results included in the PK regression process. The ratios of T_max_ and C_max_ were 0.79 ± 0.37 and 1.27 ± 0.50, respectively, probably related to the 2–3 h plasma concentration plateau without significant change.

**Figure 6:**
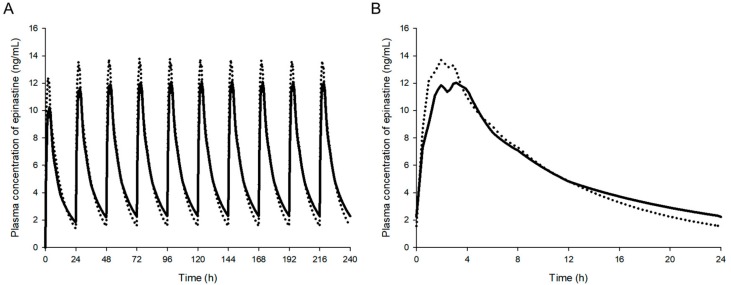
Simulation (mean value) graphs of multiple doses based on single-dose (mean) data of epinastine hydrochloride obtained using HPLC-UV (straight line) and UPLC-MS/MS (dashed line); (**A**) multiple simulation graph pattern; (**B**) estimated PK (pharmacokinetic) graph from 0 to 24 h at steady-state.

In addition, we simulated epinastine plasma concentrations at multiple doses based on single-dose data from each assay. This simulation assumed that 20 mg of epinastine hydrochloride was administered at 24 h intervals, similar to clinical dosing of once-daily (mentioned in the Section 1 Introduction), and the WinNonlin^®^ software (version 8.1, Pharsight^®^, a Certara™ Company, Princeton, NJ, USA) was used for multiple simulations. Figure 6 is a simulation (mean) graph of multiple doses based on single-dose data of epinastine hydrochloride obtained using HPLC-UV and UPLC-MS/MS, respectively. Steady-state (mean) plasma concentrations predicted by each assay were approximately 5.96–6.04 ng/mL with little difference. However, as shown in Table 6, the (mean) predicted t_1/2_ of epinastine at steady-state was 7.35 h (by UPLC-MS/MS method) and 11.35 h (by HPLC-UV method), which were quite different between the predicted values. The predicted steady-state C_max_ (mean) was 15.69 ng/mL and 13.27 ng/mL in UPLC-MS/MS and HPLC-UV method, respectively. The predicted steady-state minimum plasma concentration (C_min_) (mean) was 1.55 and 2.28 in UPLC-MS/MS and HPLC-UV analysis, respectively. There was no statistically significant difference (*p* > 0.05) in both C_max_ and C_min_ estimated from the two analytical methods. However, there was a significant difference (*p* < 0.05) in the ratio of C_max_/C_min_ to 10.12 (mean) and 5.82 (mean) for UPLC-MS/MS and HPLC-UV methods, respectively.

## 3. Discussion

Until now, few studies investigated PKs of epinastine, and analytical methods with limited validation information have been proposed. Some articles have reported bioanalytical methods for epinastine based on HPLC or specific analytical conditions [14,15,16]. However, validation information could not be confirmed in those reports. Oiwa et al. [14] reported that after oral and intravenous (IV) administration of ^14^C-epinastine hydrochloride in rats, there was dose-linearity, no gender difference on PKs, the largest distribution in the gastrointestinal tract, and a small amount excretion into the milk. Li et al. [15] revealed that there was no difference in PKs between healthy Chinese and Tibetans after a single oral administration of 20 mg of epinastine hydrochloride tablets using an HPLC-UV method. Shi et al. [16] also reported that test tablets had little difference in PK pattern from reference tablets in a bioequivalence study of epinastine hydrochloride in healthy Chinese subjects.

Several studies have used HPLC method for analysis of bulk drug [11], formulation [17], or dietary supplements [9], but are rarely used for biological sample analysis. In addition, the LLOQ values they presented were so high that it was very limited for application in quantifying biological samples. Although some researchers analyzed epinastine in rat plasma using HPLC-UV, there were common limitations of high LLOQ and long run time [12,13]. Ahirrao et al. [11] reported an HPLC-UV analysis of epinastine in bulk drug, and its LLOQ and limit of detection (LOD) were as high as 180 and 50 ng/mL, respectively. The HPLC-UV analysis of epinastine reported by Malakar et al. [17] was also for the pharmaceutical dosage form, and the LLOQ was very high as 2 μg/mL. Do et al. [9] reported the simultaneous analysis of 20 antihistamines, including epinastine, in dietary supplements, but it was very limited to be directly applied for the analysis of biological samples in consideration of different sample preparation and assay validation and long analysis time per sample. In addition, LLOQ was as high as 90 ng/mL and was not suitable for the analysis of biological samples. An HPLC-UV method, which analyzed rat plasma samples administered with 5–20 mg/kg epinastine, was introduced. However, the LLOQ of epinastine was very high, at 10 ng/mL [12]. Also, the amount of organic solvent required for preparation per sample was more than 5 mL, and the required time was very long (more than 30 min). There was also a report of rat plasma analysis, although the LLOQ value was also as high as 20 ng/mL [13]. The run-time per sample was very long, more than 16 min, and there was no assay validation, including stability, carry-over, etc.

There have also been some studies on human sample analysis using LC-MS/MS. However, they had limitations, such as high LLOQ [10], lack of detailed information on methodology [18], and much time and solvent [19]. Bae et al. [10] reported the LC-MS/MS method for epinastine in abstract form, but the LLOQ value was as high as 1 ng/mL, and specific experimental methods were lacked. In addition, Yu et al. [18] also measured epinastine concentrations in tear samples after topical ophthalmic administration of epinastine eyedrops to humans. However, due to the lack of detailed description and validation information of the assay, it was limited to apply for epinastine analysis in other biological samples. Although the analytical method reported by Shi et al. [19] was sensitive to 0.1 ng/mL of LLOQ, the required time for the analysis was very long, and the consumption of solvents was large due to HPLC method. Those previously reported contents on epinastine assay are summarized in Table S1.

Because of the clinical importance and wide application of epinastine, studies on various analytical methods and pharmacological effects have been reported from the past to the present. However, as mentioned above, there were limitations in reported epinastine assays, and so we have been working to develop UPLC-MS/MS and HPLC-UV methods that complement these limitations. As shown in our previous report [20], epinastine showed a very low C_max_ of 10–20 ng/mL. Therefore, it is essential to develop highly accurate and sensitive assay methods to obtain definite PK results, including the absorption and elimination phase, in drugs, such as epinastine.

The use of UPLC columns, optimizing the mobile phase, the use of gradient elution program, enhancing the extraction efficiency and removing impurities through appropriate sample preparation, and sample concentration through evaporation could greatly improve the sensitivity than the previously reported methods. Our results demonstrated that the developed methods were reproducible, selective, precise, accurate, and relatively impervious to endogenous interference. UPLC-MS/MS was found to be 50 times more sensitive than HPLC-UV for the determination of epinastine. Therefore, UPLC-MS/MS method was more adequate for the analysis of clinical samples where the dosages were lower. Highly sensitive UPLC-MS/MS was required in vivo in order to quantify substantially low levels of analytes in large numbers of biological samples. Thus, we quantified epinastine concentration in plasma samples obtained from all individuals. As a result, the PK profile, showing the elimination and the absorption phases, was determined accurately. In addition, UPLC-MS/MS required substantially less organic solvent and less sample volume for sample preparation. Furthermore, the required time for the total sample analysis was significantly reduced. It has, therefore, a more economical modality and is environmentally friendly. However, UPLC-MS/MS is associated with a high operating cost and is more expensive than HPLC-UV. Therefore, HPLC-UV analysis is more economical if the PK differences in HPLC-UV and UPLC-MS/MS quantification results are not as large as determined in the epinastine quantitative assay. Although the run time was 15 min, and the HPLC-UV method lasted three times longer, the HPLC-UV method was shorter than the UPLC-MS/MS method in sample preparation, suggesting that HPLC-UV method was more economical for the analysis of a small number of total samples. Our results illustrated the fact that UPLC-MS/MS could be very sensitive in comparison with HPLC-UV, which typically goes down to 1 ng/mL (LLOQ of developed HPLC-UV method). However, for general clinical studies, HPLC-UV analysis could be used, and an adequate PK profile could be obtained with a method validated between 1 and 100 ng/mL. The developed methods could be applied to the analysis of epinastine in plasma, as well as other biological samples. The reason for the difference in plasma sample preparation between UPLC-MS/MS and HPLC-UV methods is that the extraction solvent composition for each analytical instrument was developed and applied. Extraction solvents applied to HPLC-UV suspected significant matrix effects in UPLC-MS/MS, and the recovery was not satisfactory. In other words, it was optimized and applied to UPLC-MS/MS with an extraction solvent different from HPLC-UV in consideration of the matrix effect and recovery in UPLC-MS/MS.

The ratios ($\frac{UPLC-MS/MS}{HPLC-UV}$) presented in Table 5 have a very important meaning. As mentioned above (in the Section 4.5.3 Accuracy and precision), the accuracy and precision of analysis typically allowed for variability within ±15%. Variability within ±20% was allowed at the LLOQ. Nevertheless, the average ratio of V_d_/F, C_max_, T_max_, and t_1/2_ showed a difference of more than 20%. In other words, when comparing only the mean values of parameters (V_d_/F, C_max_, T_max_, and t_1/2_), they were out of 15% or 20% (usual tolerance in analysis). In the case of V_d_/F, the mean value of epinastine after a single oral dose could be calculated as low as 27% when analyzing plasma samples using UPLC-MS/MS and as short as 28% as for t_1/2_. There also could be a difference in the accumulation index as 1.117 (by UPLC-MS/MS method) and 1.301 (by HPLC-UV method), when estimating the accumulation index by the following equation: 1/1−e^−k^^·τ^, where τ is the 24 h as dosing interval. This might affect the dosing and use of epinastine in relation to a cumulative evaluation in the body at multiple doses. In the case of C_max_, the mean value of epinastine after a single oral dose could be determined as high as 27% when analyzing plasma samples using UPLC-MS/MS and as short as 21% for T_max_. This might affect the dosing and use of epinastine with regard to drug effects and toxicity. In addition, as shown in Figure 6 and Table 6, the results can be different when multiple-dose simulations are performed with single-dose PK parameters calculated based on data obtained by different assays. This could greatly affect PK evaluation. As shown in Table 6, the ratio of C_max_/C_min_ at steady-state was about 2 times greater in UPLC-MS/MS than HPLC-UV. This means that if we choose the HPLC-UV method for the calculation of epinastine PK parameters, we would be apt to underestimate the safety or toxicity of epinastine. Using the UPLC-MS/MS method, we could quantify the concentration of the epinastine terminal phase in the blood, resulting in shorter t_1/2_ (than HPLC-UV), large clearance (than HPLC-UV), and large estimation of the difference between steady-state C_max_ and C_min_ in multiple-dose simulations. In other words, the LLOQ reduction in the analysis by UPLC-MS/MS caused some differences in the estimation of PK parameters compared to HPLC-UV by quantifying epinastine elimination phases that were not accurately quantified by HPLC-UV. Our findings suggested that the choice of the assay for biosamples is critical for PK analysis and clinical dose and regimen settings. Previously reported literature [21,22] has emphasized that there are differences in the results of an assay for tacrolimus concentrations in the blood by different analytical methods. In particular, the difference between the results of microparticle enzyme immunoassay (MEIA) and LC-MS/MS was significant at low concentrations and suggested that it could significantly affect the treatment of patients [21]. In the study of Braun et al. [22], the same patient samples (tacrolimus administered) were analyzed by various analytical methods. As a result, the drug concentrations were measured differently at 10.5, 7.92, and 2.93 ng/mL in MEIA, enzyme-linked immune-sorbent assay, and LC-MS/MS. In other words, when one sample is analyzed by LC-MS/MS, the concentration is measured to be low, and the administration dose should be increased. On the other hand, when the MEIA method is used, the concentration could be over-estimated, indicating that dose adjustment is not necessary. This suggests that inappropriate treatment may have been performed. Although the therapeutic range of epinastine is not as narrow as that of tacrolimus, dose adjustment of epinastine due to differences in assays should be carefully considered in the treatment of patients, as with tacrolimus.

## 4. Materials and Methods

### 4.1. Reagents and Chemicals

**Figure 7:**
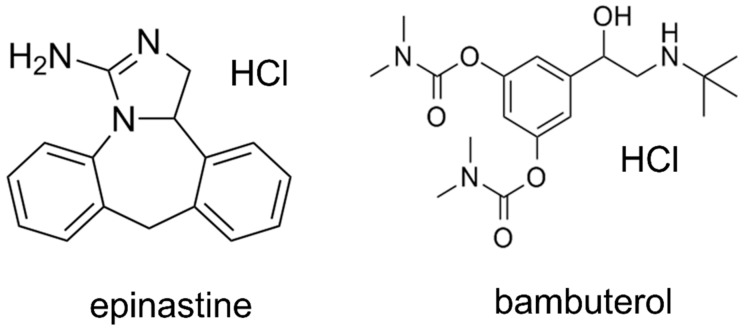
Chemical structures of epinastine hydrochloride and bambuterol hydrochloride (internal standard).

Epinastine hydrochloride (purity ≥ 99%) was obtained from Heumann Pharma GmbH & Co. Generica KG (Nuremberg, Germany), and bambuterol (purity ≥ 99%) was purchased from Sigma-Aldrich (St. Louis, MO, USA). Bambuterol was used as an IS in both methods (HPLC-UV and UPLC-MS/MS). Figure 7 presents the structures of epinastine and bambuterol. LC-MS/MS grade water (18.2 mΩ), methanol, ACN, and HPLC grade ethyl acetate were obtained from Fisher Scientific (Hampton, NH, USA). LC-MS/MS grade formic acid was purchased from DUKSAN Inc. (Ansan, Korea). All chemicals used in this study met the highest HPLC grade or quality available.

### 4.2. Chromatographic Conditions and Instrumentation

#### 4.2.1. UPLC-MS/MS Method

The UPLC-MS/MS method was conducted using an LC-30AD of Shimadzu Nexera X2 Series UPLC system (Shimadzu, Kyoto, Japan) coupled with a SIL-30AC autosampler and DGU-20A degassing unit with Shimadzu-8040 mass spectrometer. In order to obtain an optimum chromatogram, various condition tests were carried out on the mobile phase pH (0.01% formic acid in water (pH 2.7), 0.1% formic acid in water (pH 2.3), 0.05% formic acid in water (pH 2.5), 0.1% acetic acid in water (pH 2.9), 0.05% trifluoracetic acid in water (pH 2.1), and 0.3% triethylamine in water (pH 4.5), *v*/*v*), containing 1% (*v*/*v*) of 5 mM ammonium formate (pH 3.0) buffer, and column (Acquity UPLC^®^ BEH C_18_ (50 mm × 2.1 mm, 1.7 μm), HALO-C_18_ (100 mm × 2.1 mm, 2.7 μm), and Phenomenex KINETEX core-shell C_18_ (50 mm × 2.1 mm, 1.7 μm) column). The optimized chromatographic separation of epinastine was obtained on a Phenomenex KINETEX core-shell C_18_ column at an oven temperature of 40 °C. The mobile phase configuration was ACN (mobile phase B) and 0.1% aqueous formic acid, containing 1% (*v*/*v*) of 5 mM ammonium formate (pH 3.0) buffer (mobile phase A, pH 2.3), with gradient elution and a flow rate of 0.3 mL/min. The gradient elution program was as follows: 0–0.5 min (20% B), 0.5–1.5 min (20–80% B), 1.5–3.5 min (80% B), 3.5–3.51 min (80–20% B), and 3.51–5.0 min (20% B). All analytical procedures were conducted with positive electrospray ionization, and quantification was achieved using MRM modes at *m*/*z* 249.80 → 193.10 for epinastine and at *m*/*z* 367.90 → 294.20 for IS, respectively. Acquisition and analysis of data were achieved using a LabSolutions program. The injection volume was 5 μL, and the collision energies of epinastine and IS were −36 and −20 eV, respectively.

#### 4.2.2. HPLC-UV Method

The HPLC system consisted of a Shimadzu LC 10 AD series (Shimadzu, Kyoto, Japan) equipped with a photodiode array detector with LabSolutions software. A Nova-Pak C_18_ column (150 × 3.9 mm, 4 μm particle size) from Waters Inc. (Milford, MA, USA) was applied as a stationary phase, and the oven temperature was maintained at 40 °C. The mobile phase consisted of 20 mM phosphate buffer (pH 3.5) and methanol mixed with a small amount of ACN (64/30/6, *v*/*v*/*v*) at a flow rate of 0.8 mL/min. Chromatographic separation was conducted using an isocratic elution. The total run time was 15 min per sample. Injection volume was 50 μL using the Rheodyne injector, and the detection wavelength was 220 nm. Peaks were assigned by spiking the samples with standard compounds and comparison of the retention times and UV spectra.

### 4.3. Preparation of Standard Solutions

Epinastine and IS stock solutions were prepared as follows: each of epinastine hydrochloride and IS was accurately weighed and dissolved in methanol at a concentration of 1 mg/mL as epinastine prior to obtaining working solutions. All stock solutions were stored at −20 °C. The standard working solutions of epinastine (10, 20, 50, 100, 200, 500, and 1000 ng/mL in HPLC-UV analytical procedure; 0.2, 1, 10, 50, 100, 500, and 1000 ng/mL in UPLC-MS/MS analytical procedure) and the IS (100 ng/mL) were diluted stepwise with 100% methanol from the standard stock solutions. In addition, calibration standards were determined by adding each diluted working solution into a blank human plasma to obtain the final concentrations of epinastine ranging from 1–100 ng/mL in HPLC-UV analytical method and 0.02–100 ng/mL in UPLC-MS/MS analytical method. In order to examine the accuracy and precision of the developed method, QC samples of four concentration levels (1, 5, 20, and 80 ng/mL in HPLC-UV; 0.02, 5, 20, and 80 ng/mL in UPLC-MS/MS) were prepared in a similar way. Preparation of QC samples and calibration curves were performed on the same day of analysis in both methods.

### 4.4. Sample Extraction

In the HPLC-UV analytical method, epinastine was extracted from human plasma by employing the LLE method. A total of 500 μL of human plasma samples were added to a 50 μL of the IS solution (100 ng/mL of bambuterol in 100% methanol), and 500 μL of 0.1 M sodium carbonate was added to human plasma samples. Then, 4 mL of dichloromethane was added to the mixed sample and vortexed for 7 min and centrifuged at 6000× *g* for 20 min. The supernatant was removed, and the organic layer was transferred to another test tube. A 200 μL of 25 mM sulfuric acid solution was added, and the mixture was back-extracted for 1 min and centrifuged at 6000 rpm for 5 min. A 150 μL of the upper layer was transferred to an Eppendorf tube (Axygen Scientific Inc., Union City, CA, USA), and an aliquot (50 μL) of this final sample solution was taken and injected into the HPLC-UV system. In UPLC-MS/MS analytical method, the sample extraction for epinastine was extensively tested using the PP method with ACN and methanol, as well as the LLE method using di-ethyl ether, ethyl acetate, methylene chloride, and MTBE. As a result, the samples were extracted by LLE using ethyl acetate, and the protein was precipitated by PP using methanol. A total of 100 μL of human plasma samples were added to a 10 μL of the IS solution (100 ng/mL of bambuterol in 100% methanol). Then, 1000 μL of ethyl acetate-methanol (1/2, *v*/*v*) was added to the mixed samples and vortexed for 6 min and centrifuged at 13,000× *g* for 6 min. Then, 1000 μL of the supernatant corresponding to the organic layer was dried gently with a centrifugal vacuum evaporator of the CVE-3000 model (EYELA Co., Tokyo, Japan) under nitrogen gas at 35 °C for 4 h. The dried matter was reconstituted with 50 μL of 100% methanol and vortexed for 6 min. After centrifugation for 6 min at 13,000× *g*, 5 μL of aliquots were injected into the UPLC-MS/MS system.

### 4.5. Method Validation

The validation of newly developed methods was performed in accordance with the Guidance for Industry: Bioanalytical Method Validation issued by the United States FDA [23], in terms of recovery, accuracy, precision, selectivity, linearity, sensitivity, matrix effect, carryover, stability, and ISR.

#### 4.5.1. Selectivity and Sensitivity

Selectivity was evaluated to ensure the effect of endogenous substances located in the closed retention time of the analytes. Therefore, the blank plasma, zero plasma, plasma spiked with epinastine hydrochloride of LLOQ level, and real plasma samples collected after oral administration of two tablets of epinastine (epinastine hydrochloride 20 mg) to Korean subjects were used to prove and demonstrate selectivity. The used blank plasma was collected from six different individuals. The sensitivity of the method was expressed as LLOQ, LLOQ and LOD were determined as the lowest concentration of the standard samples within the range of quantification with a signal-to-noise ratio of at least 10:1 and 3:1, respectively, with an acceptable precision of less than 20% and accuracy within ±20%. All of these were evaluated using five replicate samples.

#### 4.5.2. Linearity

Calibration curves were determined using the seven calibration points by linear regression with the weighting factor of 1/concentration^2^. The linearity of the calibration curve was conducted by plotting the epinastine/IS peak area versus the theoretical concentration of epinastine. A correlation coefficient (r^2^) value with its linear calibration equation was obtained.

#### 4.5.3. Accuracy and Precision

Both intra-day accuracy and precision were assessed by analyzing the QC samples at five different times on the same day. In addition, inter-day evaluations were similarly conducted on five consecutive days. The concentration of each QC sample was quantified using the calibration standards prepared on that day. The precision was assessed by determining the CV value for the analysis of QC samples. The CV value at each concentration level was not allowed to deviate by more than ±15%. However, it was limited to 20% in LLOQ. The accuracy was determined based on the criteria, which are the mean value, not exceeding 15% of the nominal concentration. As with precision standards, it was limited to 20% in LLOQ.

#### 4.5.4. Matrix Effect and Recovery

The recovery efficiencies of epinastine were assessed for the QC samples at low, medium, and high concentrations in five replicates. In addition, the recovery efficiency of the IS was determined. The extraction recovery of the assay from human plasma was evaluated by comparing the detector (UV and MS/MS) response of extracted samples (A) with those of the samples added at the same concentration after extracting the blank plasma (B). In addition, the matrix effect for UPLC-MS/MS method was assessed by comparing the peak area of analyte post-extraction (B) from blank plasma with the absolute standard (C) of the same analyte. The matrix effect and recovery efficiency were determined by the following formula: Recovery = (A)/(B) × 100%; Matrix effect = (B)/(C) × 100%.

#### 4.5.5. Stabilities

The stabilities of epinastine in human plasma were assessed under various conditions, including freeze-thaw, long-term, and short-term stability. Two different levels of QC samples, low (5 ng/mL) and high (80 ng/mL) concentrations, were used for all stability tests. The short-term stability test was conducted by maintaining the QC samples for 24 h at 25 °C, and the long-term stability was determined by analysis of QC samples frozen for 4 weeks at −80 °C. The QC samples were stored for 24 h at −80 °C and then thawed completely at 25 °C for the freeze and thaw stability test. This cycle was repeated in succession, and analysis was conducted after the third cycle. Stabilities of epinastine and IS stock solution were measured after storage for 4 weeks at −20 °C. In addition, for post-preparative stability, the processed QC samples were placed in autosampler for 24 h at 15 °C (at UPLC-MS/MS method), and on the table for 24 h at 25 °C (at HPLC-UV method). Stabilities were determined as the % ratio of measured epinastine concentration to initial epinastine concentration (n = 5). Samples were considered stable if the test values at each level were within ±15% of the sample nominal concentration, and the precision was less than 15%.

#### 4.5.6. Carryover

The carryover test was conducted by injecting a blank sample after injecting the sample with the highest concentration (100 ng/mL for epinastine) in the standard curve. The acceptance criterion of the carryover is that the peak in the blank sample should be less than 20% of the peak in the LLOQ sample.

#### 4.5.7. Incurred Sample Reanalysis

ISR was conducted to ensure the reproducibility of newly developed methods for the analysis of epinastine. Sample selection (10% of the analyzed samples) was accomplished using a computerized random method for ISR. The selection criterion involved samples near the elimination phase in the PK profile of epinastine and C_max_. Thirty-two human plasma samples were selected and compared with initial analyzed values. The results satisfied the acceptable criteria that the variability between the mean value of the initial analysis and that of the reanalysis was within ±15%. In addition, reanalysis values for 67% of all samples were within 20% of their initial values.

### 4.6. Pharmacokinetic Studies

Twenty-six healthy males of Korean subjects (22–25 years of age, bodyweight 60–85 kg, height 165–195 cm) were recruited for a clinical trial (as bioequivalence test). The Institutional Review Board of the Institute of Bioequivalence and Bridging Study (Chonnam National University, Gwangju, Korea) approved this study protocol (Bioequivalence Test No. 324; 02.04.2005). The clinical trial was conducted according to the revised Declaration of Helsinki for biomedical research involving human subjects and the rules of Good Clinical Practice. The written consent was obtained prior to participating from all participants. In addition, all participants received laboratory, physical, and medical tests prior to a clinical trial. As a result, this study included only well-healthy participants. All the participants fasted more than 10 h before receiving 20 mg epinastine hydrochloride tablets followed by fasting for 4 h. In addition, they avoided drinks or foods containing caffeine or xanthine ingredients during this study. All participants received two tablets of epinastine (epinastine hydrochloride 20 mg) with water of 240 mL. The blood samples were taken from the forearm vein before the administration (0 h) and 0.5, 1, 1.5, 2, 2.5, 3, 4, 6, 8, 12, and 24 h after oral administration. The blood samples were transferred into Vacutainer^®^ tube (Becton, Dickinson and Company, Franklin Lakes, NJ, USA) of 10 mL capacity and were centrifuged (10,000 × *g*) immediately for 10 min at 4 °C. The supernatant plasma samples were transferred to polyethylene tube and stored at −80 °C until further analysis. T_max_, C_max_, and C_min_ were determined using the plasma drug concentration curve over time. The AUC_0–∞_ was integrated by a linear trapezoidal rule to the final measured concentration (C_last_) and extrapolated to infinity by adding the area from C_last_ (AUC_0–t_) to infinity (C_last_/k); k means the elimination rate constant at terminal phase. The CL/F was determined by dividing the dose of epinastine by the AUC_0–∞_, where F is the bioavailability of oral administration. The t_1/2_ was determined as 0.693/k and V_d_/F as dose/k·AUC_0–∞_. All PK parameters were determined via noncompartmental analysis using the WinNonlin^®^ software (version 8.1, Pharsight^®^, a Certara™ Company, Princeton, NJ, USA).

### 4.7. Pharmacokinetic Parameters Comparison

In addition to those mentioned (in the Section 4.8 Statistical analysis) to compare the differences in PK parameter values according to the analytical methods, the parameter values calculated using HPLC-UV and UPLC-MS/MS were divided as follows: $\frac{PK\;parameters\;by\;UPLC-MS/MS}{PK\;parameters\;by\;HPLC-UV}$. Closer values to 1 mean that there is little effect on the PK parameters between the two methods.

### 4.8. Statistical Analysis

Statistical analysis was performed using the Statistical Package for the Social Sciences (SPSS) software version 23 (IBM, Armonk, NY, USA) on the PK parameters calculated by HPLC-UV and UPLC-MS/MS methods. In other words, all PK parameters determined by each quantification method were analyzed for statistical significance by Student’s t-test with *p* < 0.05, indicating a significant difference.

## 5. Conclusions

The purposes of this study were to compare the PK parameter values of epinastine obtained using the two methods and to discuss their meanings, as mentioned in the introduction. In this study, we provided the newly developed analysis methods—UPLC-MS/MS and HPLC-UV. Our study focused on comparing the performances of newly developed UPLC-MS/MS and HPLC-UV methods for the determination of epinastine in human plasma.

No published report has compared the differences in analytical methods, such as HPLC-UV and UPLC-MS/MS, in biosamples for epinastine. Generally, UPLC-MS/MS methods show higher throughput, selectivity, and sensitivity compared to HPLC-UV methods [24]. However, because MS detectors are expensive, analyzing biosamples using relatively less expensive UV detectors would be a great economic advantage. Numerous studies comparing LC-MS and LC-UV methods in the analysis of biosamples for other drugs have been reported from the past [24,25,26,27,28,29,30]. The purpose of the inter-comparison of these methods is perhaps to ensure that the results are identical even if one of the two methods is used. In other words, by choosing the appropriate analytical method for a specific situation, it would be possible to derive the optimal PK result. In general, the use of LC-UV is more appropriate where rapid turn round of data is not required, and plasma concentrations are high. However, if sensitivity is an issue, limited amounts of plasma are available, and/or where tight deadlines are pivotal, LC-MS is the method of choice [30].

Both methods were fully validated according to international bioanalytical method guidelines and represented a useful tool for the characterization of PKs of 20 mg epinastine hydrochloride tablets in Korean subjects. Furthermore, comparison (especially focused on PK parameters) of two commonly used analytical methods (HPLC-UV and UPLC-MS/MS) for in vivo analysis of epinastine was performed for the first time. As a result, this method comparison study confirmed that different dose and usage settings might be possible based on PK parameters calculated by other methods. Therefore, if careful consideration is required for dose and regimen settings of drugs, it may be necessary to consider differences in interpretation of PKs according to the assays reported in this study. Finally, our conclusion was that the choice of the assay was very important for the analysis of biological samples (especially where the concentration of drug in the plasma sample is relatively low, as in this study). The UPLC-MS/MS method, which could provide a lower LLOQ than the HPLC-UV method, quantified the concentrations of the drug’s initial absorption and elimination phases in the blood, resulting in more clear PK profiles in case of epinastine. However, this conclusion could not be generalized for some drugs. Due to the presence of the matrix effect or the reduction of ionization sites, there might be some drugs that are more sensitively quantified in LC-UV than LC-MS/MS. These results might affect the calculation of PK parameters (such as t_1/2_ and clearance) and simulation results at multiple doses, although there were no significant differences in concentration values. In the case of drugs requiring tight therapeutic drug monitoring (TDM), very careful attention should be paid to the dosage and regimen based on these PK parameters. As a result, this study suggested that UPLC-MS/MS was better for PK studies on substances with low plasma concentrations despite the maximum daily dose of the drug, such as epinastine. Our findings are very new and have not been reported previously and are expected to be very important data for PK studies and interpretation of results.

## Figures and Tables

**Table 1:** Precision and accuracy of UPLC-MS/MS and HPLC-UV analysis for the determination of epinastine in human plasma (Mean ± SD, *n* = 5).

Method	Spiked Conc. (ng/mL)	Intra-Batch (*n* = 5)			Inter-Batch (*n* = 5)		
		Measured Conc. (ng/mL, Mean ± SD)	Precision (CV, %)	Accuracy (%)	Measured Conc. (ng/mL, Mean ± SD)	Precision (CV, %)	Accuracy (%)
UPLC-MS/MS	QC 0.02	0.020 ± 0.001	4.90	102.00	0.019 ± 0.001	5.07	94.50
	QC 5	4.918 ± 0.067	1.32	98.36	5.115 ± 0.085	1.66	102.30
	QC 20	19.386 ± 0.924	4.48	96.93	18.976 ± 1.373	6.53	94.88
	QC 80	82.029 ± 4.285	5.22	102.54	84.281 ± 5.249	6.23	105.35
HPLC-UV	QC 1	1.108 ± 0.107	11.63	110.23	1.036 ± 0.113	4.04	114.47
	QC 5	4.866 ± 0.533	12.52	95.64	5.158 ± 0.114	2.67	106.23
	QC 20	21.043 ± 1.001	3.16	105.07	18.863 ± 1.961	6.09	93.93
	QC 80	76.121 ± 7.473	9.34	95.15	82.421 ± 6.582	8.23	103.03

CV, coefficient of variation; QC, quality control.

**Table 2:** Recovery and matrix effect for the determination of epinastine in human plasma via UPLC-MS/MS and HPLC-UV methods (Mean ± SD, n = 5).

Method	Spiked Conc. (ng/mL)	Measured Conc. (ng/mL, Mean ± SD)	Recovery (%)	Measured Conc. (ng/mL, Mean ± SD)	Matrix Effect (%)
UPLC-MS/MS	QC 5	4.88 ± 0.16	97.62 ± 3.14	4.76 ± 0.07	95.28 ± 1.42
	QC 20	19.19 ± 0.57	95.93 ± 2.85	19.33 ± 0.49	96.63 ± 2.44
	QC 80	76.99 ± 3.46	96.24 ± 4.33	76.57 ± 1.68	95.71 ± 2.10
	IS (bambuterol) 10	9.95 ± 0.35	99.46 ± 3.54	9.74 ± 0.32	97.35 ± 3.16
HPLC-UV	QC 5	4.58 ± 0.26	91.58 ± 5.11	-	-
	QC 20	18.48 ± 1.05	92.40 ± 5.27	-	-
	QC 80	75.49 ± 5.11	94.36 ± 6.39	-	-
	IS (bambuterol) 10	9.69 ± 0.47	96.94 ± 4.73	-	-

IS, internal standard.

**Table 3:** Stabilities of epinastine under various conditions of UPLC-MS/MS and HPLC-UV quantification methods (Mean ± SD, n = 5).

Method	Spiked Conc. (ng/mL)	Short-Term Stability (24 h)	Long-Term Stability (4 weeks)	Freeze-Thaw Stability (3 cycles)	Post-Preparative Stability (24 h)
UPLC-MS/MS	QC 5	99.44 ± 3.11	101.53 ± 1.49	101.62 ± 2.66	99.33 ± 4.02
	QC 80	98.21 ± 5.22	98.51 ± 0.99	98.11 ± 3.32	102.09 ± 1.48
HPLC-UV	QC 5	97.91 ± 4.02	98.42 ± 3.84	100.10 ± 4.35	98.13 ± 2.62
	QC 80	100.35 ± 4.83	99.55 ± 3.67	96.04 ± 4.93	101.91 ± 3.17

**Table 4:** Summary of information on UPLC-MS/MS and HPLC-UV methods developed in this study.

	UPLC-MS/MS	HPLC-UV
LLOQ	0.02 ng/mL	1 ng/mL
LOD	0.007 ng/mL	0.3 ng/mL
Calibration range	0.02–100 ng/mL	1–100 ng/mL
Run time	5 min	15 min
Sample extraction	LLE with ethyl acetate and PP with methanol	LLE with dichloromethane
Sample volume	100 μL	500 μL
Column type and size	KINETEX core-shell C_18_ (50 × 2.1 mm, 1.7 μm particle size; Phenomenex, USA)	Nova-Pak C_18_ (150 × 3.9 mm, 4 μm particle size; Waters, USA)
Mobile phase	0.1% aqueous formic acid:5 mM ammonium formate:ACN (1/1/4, *v*/*v*/*v*)	20 mM phosphate buffer:methanol:ACN (64/30/6, *v*/*v*/*v*)
Flow rate	0.3 mL/min	0.8 mL/min
Injection volume	5 μL	50 μL
Internal standard	Bambuterol (10 ng/mL)	Bambuterol (10 ng/mL)
Results	All plasma samples were quantified in the range (even in the 24 h samples).	Some plasma samples were not quantified within the range and showed values below LLOQ (at 0.5 h of initial absorption of some samples and 24 h of post-elimination).

LLOQ, lower limit of quantitation; LOD, limit of detection; LLE, liquid-liquid extraction; PP, protein precipitation.

**Table 5:** Pharmacokinetic parameters of epinastine in humans after oral administration of 20 mg epinastine hydrochloride tablet in UPLC-MS/MS and HPLC-UV quantification methods (Mean ± SD).

Parameter	UPLC-MS/MS	HPLC-UV	*p*-Value	$\mathrm{Ratio}\;(\frac{UPLC-MS/MS}{HPLC-UV})$
AUC_0–∞_ (h·ng/mL)	144.88 ± 45.86	143.01 ± 44.43	0.98	1.01 ± 0.32
AUC_0–t_ (h·ng/mL)	129.84 ± 39.21	115.21 ± 34.30	0.78	1.13 ± 0.36
CL/F (L/h)	150.99 ± 46.05	156.72 ± 65.34	0.92	0.96 ± 0.33
V_d_/F (L)	1519.50 ± 546.31	2091.30 ± 588.22	0.45	0.73 ± 0.26
C_max_ (ng/mL)	14.82 ± 6.06	11.62 ± 3.63	0.47	1.27 ± 0.50
T_max_ (h)	2.13 ± 1.20	2.71 ± 1.27	0.52	0.79 ± 0.37
t_1/2_ (h)	7.08 ± 1.80	9.95 ± 3.00	0.41	0.72 ± 0.21

AUC_0–∞_, area under the curve; AUC_0–t_, area to final measured concentration; CL/F, clearance; V_d_/F, volume of distribution; C_max_, the maximum plasma concentration; T_max_, the time to reach C_max_; t_1/2_, half-life.

**Table 6:** Estimated pharmacokinetic parameters of epinastine in humans at steady-state after oral multiple administration of 20 mg epinastine hydrochloride tablet in UPLC-MS/MS and HPLC-UV quantification methods (Mean ± SD).

Parameter	UPLC-MS/MS	HPLC-UV	*p*-Value	$\mathrm{Ratio}\;(\frac{UPLC-MS/MS}{HPLC-UV})$
AUC_0–∞_ (h·ng/mL)	157.38 ± 56.89	181.18 ± 68.22	0.77	0.87 ± 0.31
AUC_0–t_ (h·ng/mL)	139.75 ± 45.15	139.80 ± 43.56	0.99	1.00 ± 0.32
CL/F (L/h)	141.10 ± 44.41	131.69 ± 71.45	0.88	1.07 ± 0.33
V_d_/F (L)	1457.58 ± 484.22	1878.28 ± 486.91	0.51	0.78 ± 0.26
C_max_ (ng/mL)	15.69 ± 6.17	13.27 ± 3.89	0.62	1.18 ± 0.46
C_min_ (ng/mL)	1.55 ± 0.83	2.28 ± 1.08	0.35	0.68 ± 0.36
C_max_/C_min_	10.12 ± 3.98	5.82 ± 1.71*	0.01	1.74
T_max_ (h)	2.02 ± 1.16	2.65 ± 1.28	0.46	0.76 ± 0.43
t_1/2_ (h)	7.35 ± 1.78	11.35 ± 4.33*	0.04	0.65 ± 0.16

* *p* < 0.05, compared with calculated parameters by UPLC-MS/MS method.

## Supplementary Materials

The following are available online at https://www.mdpi.com/1420-3049/25/1/209/s1, Figure S1: Calibration curves of epinastine in human plasma by HPLC-UV (A) and UPLC-MS/MS methods (B). The linear straight line refers to the regression line and the vertical bars represent the standard deviation of the mean (n = 5)., Table S1: Summary of previously reported epinastine assays.
