# Supplementary material for: Pharmacokinetic Comparison of Epinastine Using Developed Human Plasma Assays
Source: Molecules. 2020 Jan 3;25(1):209. doi: 10.3390/molecules25010209 (PMC6983027; doi:10.3390/molecules25010209)
Supplement: Supplementary file 1 [file molecules-25-00209-s001.pdf]

Table S1. Summary of previously reported epinastine assays.

	HPLC-UV							LC-MS/MS			
**References**	Ahirrao et al. 2012	Malakar et al. 2012	Li et al. 2011	Shi et al. 2007	Ogiso et al. 2001	Ohtani et al. 1996	Oiwa et al 1992	Shi et al. 2016	Do et al. 2015	Bae et al. 2009	Yu et al. 2005
**Sample**	Bulk drug	Formulation	Human plasma	Human plasma	Rat plasma	Rat plasma	Rat plasma	Human plasma	Dietary supplements	Human plasma	Human tear
**Calibration range**	-	2-200 μg/mL	-	-	-	20-1000 ng/mL	-	0.1-40 ng/mL	0.1-5 μg/mL	-	-
**LOD**	50 ng/mL	0.2 μg/mL	-	-	-	10 ng/mL	-	-	0.03 μg/mL	-	-
**LOQ or LLOQ**	180 ng/mL	0.5 μg/mL	-	-	10 ng/mL	20 ng/mL	-	0.1 ng/mL	0.09 μg/mL	1 ng/mL	-
**Run time**	25 min	10 min	-	-	30 min	16 min	-	-	10 min	4 min	-
**Limitations**	Relatively long analysis time per sample	Limited to be directly applied for analysis of biological samples	Lack of specific assay information and validation data	Lack of specific assay information and validation data	Lack of specific assay information and validation data	Limited to be directly applied for analysis of biological samples	Lack of specific assay information and validation data	Required time for analysis and consumption of solvents were large due to HPLC method	Limited to be directly applied for analysis of biological samples	Lack of specific assay information and validation data	Lack of specific assay information and validation data

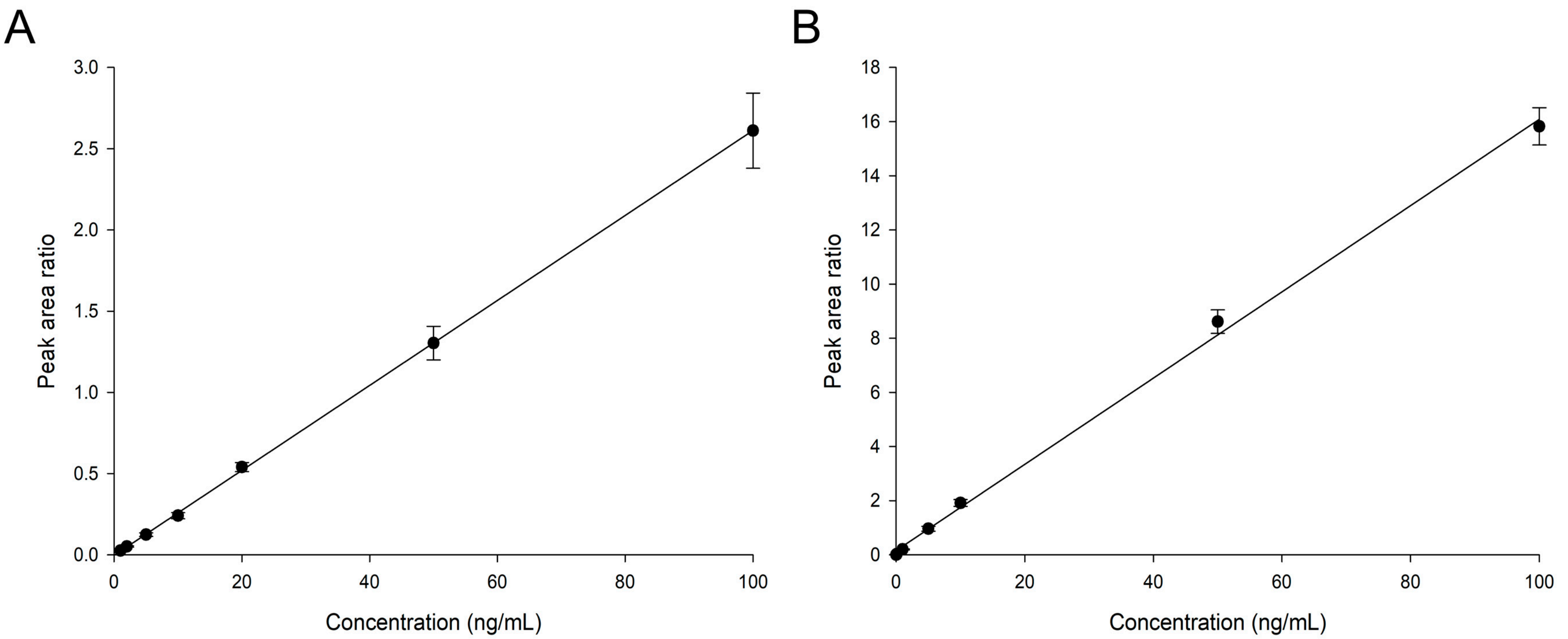

Figure S1. Calibration curves of epinastine in human plasma by HPLC-UV (A) and UPLC-MS/MS methods (B). The linear straight line refers to the regression line and the vertical bars represent the standard deviation of the mean (n = 5).
